# Is alkaline phosphatase the smoking gun for highly refractory primitive leukemic cells?

**DOI:** 10.18632/oncotarget.12497

**Published:** 2016-10-06

**Authors:** Laura G. Rico, Jordi Juncà, Mike D. Ward, Jolene Bradford, Jordi Petriz

**Affiliations:** ^1^ Josep Carreras Leukemia Research Institute, Barcelona, Spain; ^2^ Thermo Fisher Scientific, Eugene, Oregon, USA

**Keywords:** alkaline phosphatase, stem cells, leukemic stem cells, CD34, Vybrant DyeCycle Violet

## Abstract

With the aim to detect candidate malignant primitive progenitor populations, we modified an original alkaline phosphatase (ALP) stem cell detection method based on the identification of alkaline phosphatase fluorescent cells in combination with flow cytometry immunophenotyping. Over a period of one year, we have been using this technique to study its activity in patients with leukemia and lymphoma, showing that changes in the alkaline phosphatase levels can be used to detect rare populations of highly refractory malignant cells. By screening different blood cancers, we have observed that this activity is not always restricted to CD34+ leukemic cells, and can be overexpressed in CD34 negative leukemia. We have verified that this method gives accurate and reproducible measurements and our preliminary results suggest that CD34+/ALP^high^ cells appear to sustain leukemogenesis over time.

## INTRODUCTION

Alkaline phosphatase (ALP) is a 140 kD enzyme with a dimeric structure and is capable of binding Zn^2+^ and Mg^2+^ ions at different sites to stimulate or inhibit its catalytic reaction. In humans, four forms of alkaline phosphatase cDNA have been cloned: one of them restricted to the intestine [1], one restricted to the placenta [2], one restricted to germ cells and teratomas [3], and the other widely distributed (liver, bone, kidney) [4] [5], where it functions to hydrolyze phosphate groups from a wide spectrum of physiological substrates. ALP was originally described in a histochemical study as a marker for various tissues, especially as related to bone formation in osteogenic mouse tumors [6]. The authors used a technique described by Takamatsu [7] and independently by Gomori [8], based on the deposition of calcium phosphate at the site of enzyme action. In 1973, ALP was reported as a histochemical marker for embryonal carcinoma cells with two different alkaline phosphatases localized in stem cell populations and in embryonic ectodermal cells [3]. ALP is used as a marker for pluripotent stem cells, embryonic stem cells, induced pluripotent stem cells and embryonic germ cells [9]. ALP is highly elevated in these cells and western blot, ELISA, and chromogenic substrates in combination with immunohistochemistry are commonly used to assay these levels. More recently, alkaline phosphatase activity has been analyzed using highly sensitive fluorescent and chemiluminescent substrates, in both fixed and living cells [10].

Alkaline phosphatase activity is altered in some disease states. Neutrophil ALP activity increases in patients with bacterial infection. Studies have been extended to ALP activity in polycythemia vera [11], chronic myelogenous leukemia [12] and paroxysmal nocturnal hemoglobinuria [13]. Alkaline phosphatase has also been shown as a useful cytochemical marker for canine lymphocyte subsets and some types of lymphoma and lymphoid leukemia [14], and more recently it has been used for acute myeloid leukemia (AML) confirmation in dogs, opening new opportunities for healthcare innovation in canine disease [15].

Blood cancers are characterized by different symptoms, types, stages, and treatments. Acute lymphoblastic leukemia (ALL) is a clonal aggressive malignancy of the hematopoietic system that can progress quickly, and if not treated can cause the death of the patient in few months. ALL is the most common blood cancer in children and is the leading cause of death before 20 years of age [16]. High leukocyte counts at diagnosis are predictive of poor prognosis, but these counts have limited prognostic relevance for ALL subtypes. The time to achieve complete remission or time to clearance of malignant cells measured as an early response to treatment is extremely powerful for the prognosis of ALL in children. Flow cytometry immunophenotyping is used to classify ALL subgroups in T-cell or B-cell ALL as well as for the study of minimal residual disease in combination with molecular biology. These are critical for studying aberrant marker expression of cell surface antigens and genetic alterations, such as the rearrangement of *MYC* and *MLL*. Some ALL subtypes originate in the stem cell compartment and are more aggressive than others and thus require, if possible, the early detection of clonogenic stem cells to prevent the expansion by means of aggressive treatment intervention.

## RESULTS AND DISCUSSION

With the aim of identifying primitive leukemic progenitor cells in human leukemia, we modified an alkaline phosphatase stem cell detection method based on determination of a fluorescent alkaline phosphatase substrate in cells positive for the enzyme (ALP+). The original method using fluorescence microscopy [10] was adapted for flow cytometric measurements. The alkaline phosphatase live stain (APLS) is a cell-permeable fluorescent substrate for ALP that is non-toxic to cells, diffusing out over the course of two hours (Supplementary information I). We have been using this protocol to study the alkaline phosphatase activity in human leukemia and lymphoma. Here we show one-year history from a follow-up of the same patient, a 27-year-old female diagnosed in May 2010 with B common acute lymphoblastic leukemia with 46, XX normal karyotype (Case 1). Reverse transcription-polymerase chain reaction never detected the presence and expression of p190 and p210 BCR-ABL1 fusion transcripts. Moreover, neither the p210 (b3a2, b2a2, b3a3, b2a3) nor p190 (e1a2, e1a3) isoforms were detected. Interphase FISH was negative for rearrangements of 11q23/MLL, CDKN2A/p16(9p21), and TCF3/PBX1. Antigen expression by multiparameter flow cytometry immunophenotyping at diagnosis gave the following profile: 10% MPO, 85% cytoplasmic CD79a, 10% cytoplasmic CD3, 42% TdT, 1% cytoplasmic μ chain, 88% CD34, 95% CD58 (87% coexpressed with CD19), 70% CD66c, and 18% CD33. Interestingly, CD66c expression was restricted from 5 to 10% of blast cells at second and third relapse respectively, whereas CD33 expression increased up to the 60%. Antigen expression was evaluated at first relapse (November 2012), showing 40% CD34+ blast cells with identical phenotype of refractory disease as compared with diagnosis (Supplementary information II).

**Figure 1:**
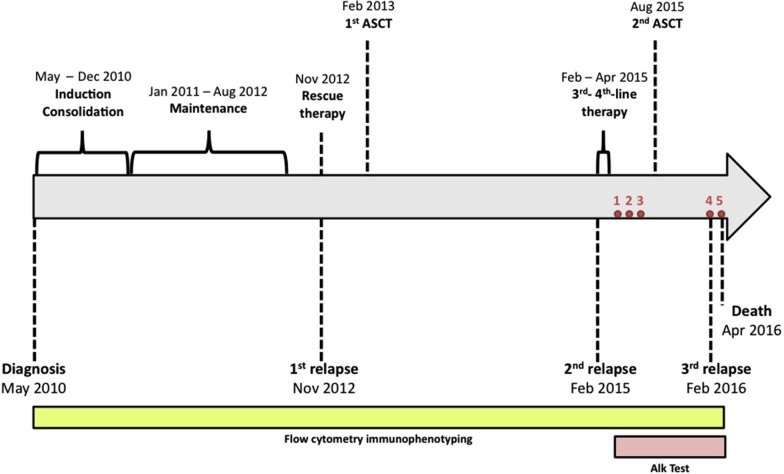
The timeline represents treatments and interventions, and the patient's clinical state, respectively The patient was initially treated with an induction therapy based on the PETHEMA-LAL-AR/03 protocol for therapy of high risk ALL, a consolidation therapy in blocks of two cycles, and maintenance therapy. After first relapse, the patient received a rescue therapy with FLAG-IDA (fludarabine, cytarabine, idarubicin and G-CSF) and then received an allogeneic transplant. After 2 years, the patient suffered a second relapse and received a third- and fourth-line therapy (vincristine, prednisone, daunorubicin and inotuzumab ozogamicin). The patient received a second allogeneic transplant and suffered a third relapse. On March 2016 the patient had fever and pneumonia and was treated with antibiotics, but her condition worsened dramatically and she finally died on April 3rd, 6 years after diagnosis. Red circles indicate the bone marrow flow cytometry analyses to detect alkaline phosphatase activity (ALP Test) in combination with CD34+ staining. The ALP Test was adapted from previously described methods and only was used to determine the alkaline phosphatase activity when was internally validated.

On February 2015, the patient had a second relapse with 88% of blast cells (morphology of lymphoid-like cells), and received a third line treatment (vincristine, prednisone and daunorubicin) that failed with remaining blast cells after 2 weeks. From March to April 2015, the patient received a new treatment (methotrexate/cytarabine) that failed again, and she completed a new treatment with inotuzumab ozogamicin, achieving complete remission (MRD negative). On August 2015, the patient received a second allogeneic transplantation. Marrow aspirates post-transplant were compatible with cytological remission, 0.0044% MRD and 100% chimerism (first month); cytological remission, 0.038% MRD and 100% chimerism (third month). Cell cycle analysis of five different marrow aspirates, as annotated in Figure 1, gave the following results: 1) %G1 = 92.5; %S = 1.97; %G2 = 6.27, 2) %G1 = 84.1; %S = 16; %G2 = 0.844, 3) %G1 = 89.7; %S = 8.56; %G2 = 1.88, 4) %G1 = 94; %S = 6.5; %G2 = 0.9, 5) %G1 = 80.2; %S = 19; %G2 = 1.79. Ploidy analysis showed diploid pattern (DNA index = 1) (Supplementary information II).

**Figure 2:**
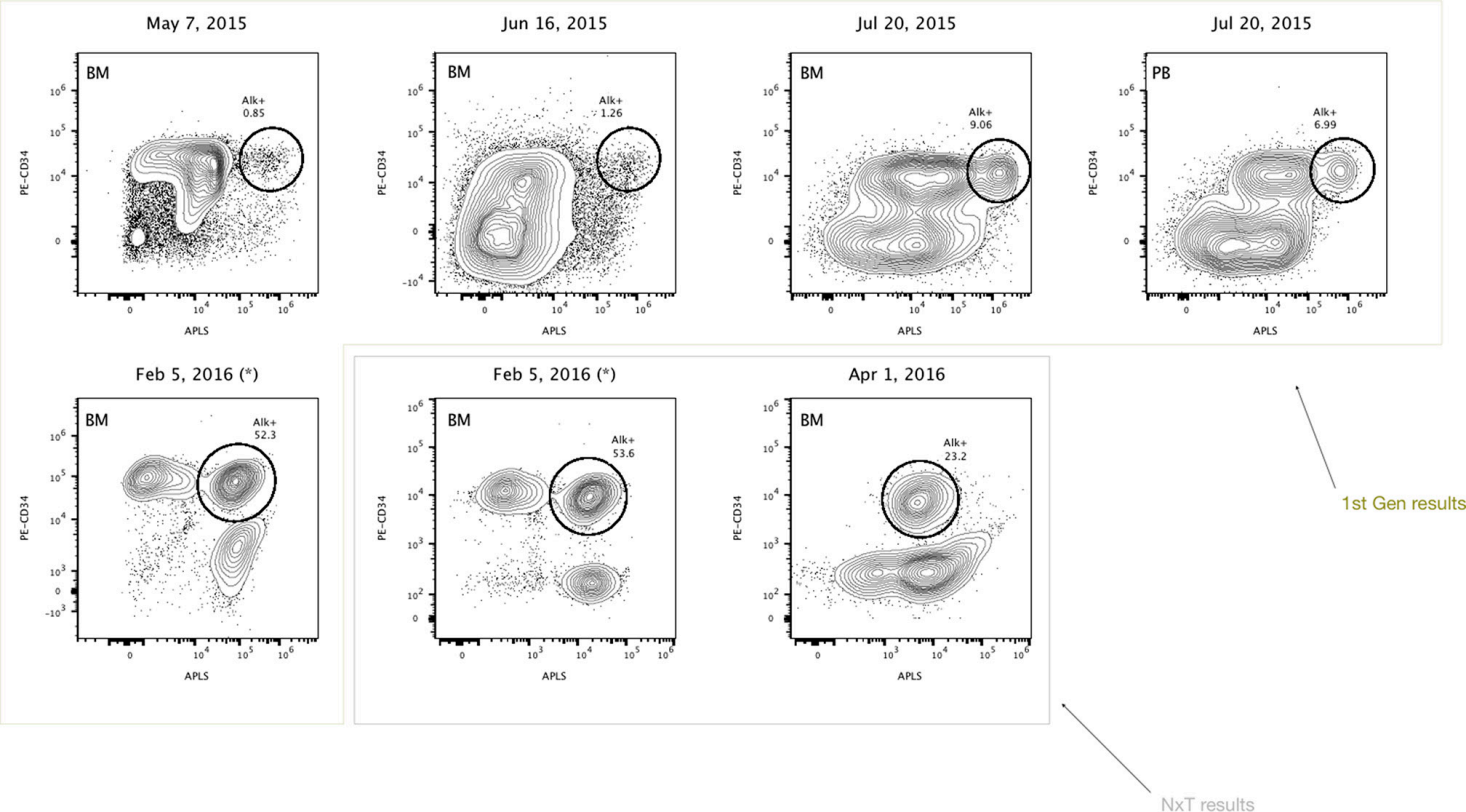
Highly refractory B-cell acute lymphoblastic leukemic CD34+ cells expressing high levels of alkaline phosphatase activity (Case 1) Reference contour plots for five bone marrow (BM) aspirates and one peripheral blood (PB) analysis obtained in the last year of the follow-up, showing the performance of the alkaline phosphatase test in combination with CD34 staining. Prospective comparison and classification of ALP+ cells from new independent bone marrow samples corresponding to the same B-ALL patient, show different subsets of CD34+ cells. Each individual circle represents CD34+ cells with high expressing levels of alkaline phosphatase activity, presumably enriched in primitive refractory cells. Comparison of well-defined encircled populations consisting of CD34+/ALP high cells provides valuable information for dual parameter contour plots over time, to better discriminate subsets of apparently clonal CD34+ cells. First generation results data were obtained using blue laser excitation whereas NxT data were obtained using dual-laser excitation on the Attune Acoustic Focusing Cytometer and the Attune NxT Cytometer respectively (Thermo Fisher Scientific). Unlysed blood samples were simultaneously stained with PE-CD34, APLS, and DCV. The filter combination used consisted of 555 DLP, 530/30 (APLS), and 620 DLP, 574/26 (PE). For the Attune NxT, PE-CD34 was excited with the yellow laser at 561 nm and its emission was collected using the following filter combination: 595 LP, 600 DLP, and 585/16. APLS, PE and DCV fluorescence were displayed in logarithmic scale.

**Figure 3:**
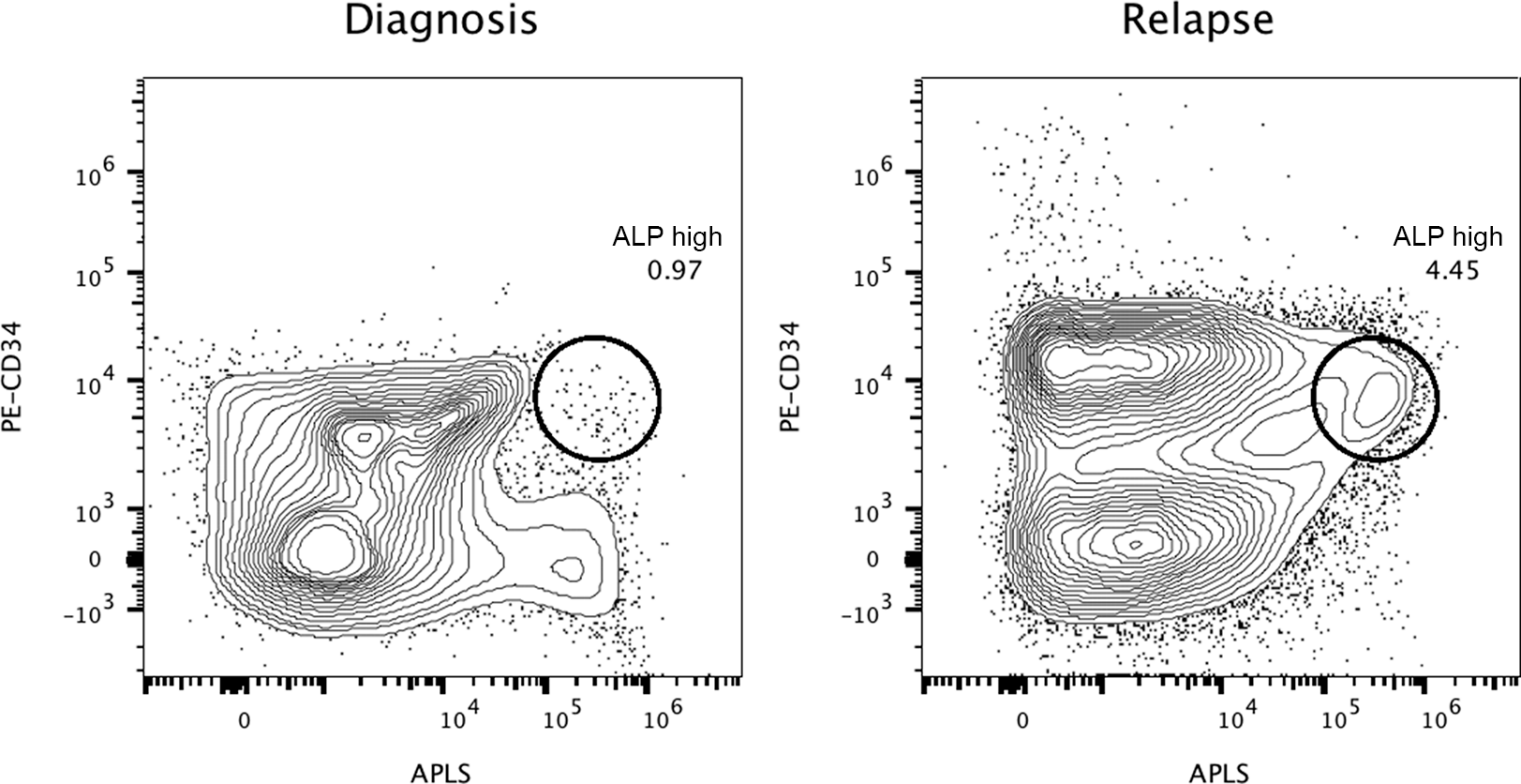
Highly refractory B-cell acute lymphoblastic leukemic CD34+ cells expressing high levels of alkaline phosphatase activity at diagnosis and relapse (Case 2) Reference contour plots for two bone marrow aspirates obtained from the second patient, showing the performance of the alkaline phosphatase test in combination with CD34 staining. Prospective comparison and classification of ALP+ cells also shows different subsets of CD34+ cells. Each individual circle represents CD34+ cells with high expressing levels of alkaline phosphatase activity, presumably enriched in primitive refractory cells. Comparison of well-defined encircled populations consisting of CD34+/ALP high cells provides valuable information at the moment of diagnosis and similarly at relapse, six months later.

On February 2016 the patient had a third cytological relapse (82% of blast cells with B-ALL phenotype), receiving a new treatment with corticoids, inotuzumab ozogamicin and vincristine. On March 2016, fever and pneumonia were treated with antibiotics, but the patient's condition worsened dramatically. She finally died on April 3^rd,^ 6 years after diagnosis. The timeline of the Case 1 with diagnostic and therapeutic events is displayed in Figure 1. The ALP assay was internally validated and was used over the last year of treatment. Five bone marrow aspirate specimens were taken from May 2015 to April 1st 2016. In parallel, the fourth aspirate was compared with peripheral blood analysis (Figure 2). By using the alkaline phosphatase assay, our results suggest that apparently clonal leukemic refractory CD34+ cells can be classified into functional states based upon the different activity levels of the phosphatase. If true, the main differences in the activity of the enzyme, accordingly with previous observations showing that primitive stem cells express the highest phosphatase activity [10–17], could help to identify and differentiate new oligoclonal/pseudoclonal populations in patients with neoplastic malignancies. Preliminary results obtained in our laboratory have shown that alkaline phosphatase can be expressed at high levels in leukemia. We also analyzed two independent bone marrow aspirates at diagnosis and relapse from a 66-year-old female diagnosed in May 2015 with B common acute lymphoblastic leukemia, 54, XX hyperdiploid karyotype (Case 2). RT-PCR never detected the presence and expression of BCR-ABL1 fusion transcripts and interphase FISH was negative for rearrangements of 11q23/MLL. Flow cytometry immunophenotyping was positive for: PAX5, CD20, CD34, TdT, CD10 and CD99. FCM was negative for CD3, CD5, cyclin D1 and BCL6 (Supplementary information III). The patient was enrolled in the PETHEMA LAL-07OLD protocol and six months later had a first cytological relapse. The patient received a new chemotherapy with vincristine and prednisone for two weeks, followed by blinatumomab. After treatment, minimal residual disease (MRD) was determined by searching cells with the abnormal phenotype detected at the moment of diagnosis (CD10/CD123/CD19/CD20/CD34) through a live-gate strategy. The final result was that this phenotype was detected in 0.02% of the total bone marrow population. However, the patient relapsed and died three months later. In Figure 3, we show the performance of the alkaline phosphatase test in combination with CD34 staining in the second patient, with CD34+/ALP^high^ cells at diagnose and relapse.

**Figure 4:**
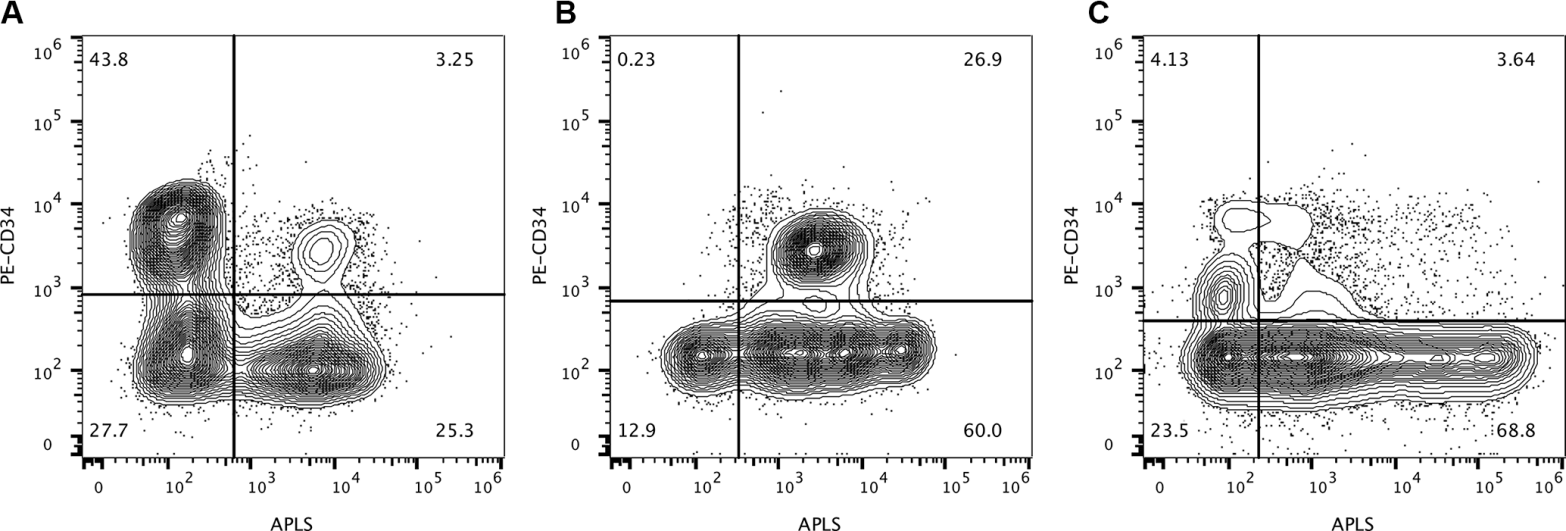
Alkaline phosphatase activity is not restricted to CD34-positive cells Figure (**A**) represents the alkaline phosphatase test of a bone marrow aspirate from a 61-year-old patient with myelodysplastic syndrome developed into acute myeloid leukemia. The time and date of sample collection was at diagnosis. Figure (**B**) represents a bone marrow aspirate from a 78-year-old patient with refractory anemia with excess blasts (RAEB-2) developed into acute myeloid leukemia at diagnosis, and Figure (**C**) shows the APL test on a marrow aspirate from a 12-year-old patient with intermediate risk B-cell precursor acute lymphoblastic leukemia during maintenance therapy leaving an undetectable minimal residual disease.

We have observed that this activity is not always restricted to CD34+ leukemic cells but can be overexpressed in CD34 negative leukemic blasts from independent specimens (Figure 4). Figure 4A represents the ALP test of a bone marrow aspirate from a 61-year-old patient with myelodysplastic syndrome developed into acute myeloid leukemia. The time and date of sample collection was at diagnosis. Figure 4B represents a bone marrow aspirate from a 78-year-old patient with refractory anemia with excess blasts (RAEB-2) developed into acute myeloid leukemia at diagnosis, and Figure 4C shows the APL test on a marrow aspirate from a 12-year-old patient with intermediate risk B-cell precursor acute lymphoblastic leukemia during maintenance therapy leaving an undetectable MRD.

Hence, it is hypothesized that this assay could help to dissect a functional phosphatase-based hierarchy originated from a very primitive hematopoietic cell. Furthermore, we have shown that the ALP substrate used in this assay is not effluxed by the ATP Binding Cassete G2 (ABCG2) transporter and that alkaline phosphatase activity is increased in Side Population cells when glioblastoma-derived cells are cultured at physiological oxygen concentrations (5% O_2_) and compared with cells maintained at atmospheric oxygen concentration (21% O_2_) (Supplementary information IV).

**Figure 5:**
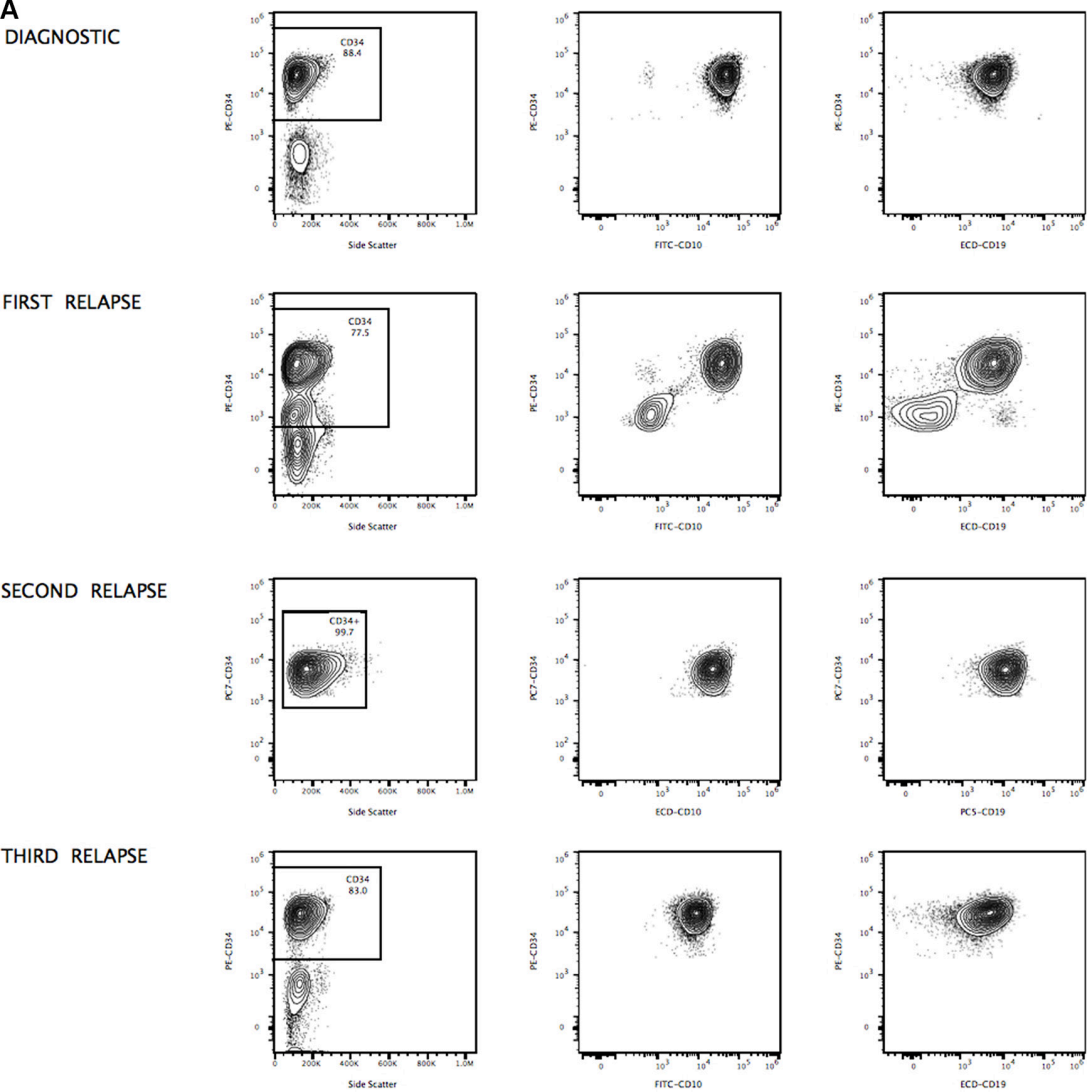

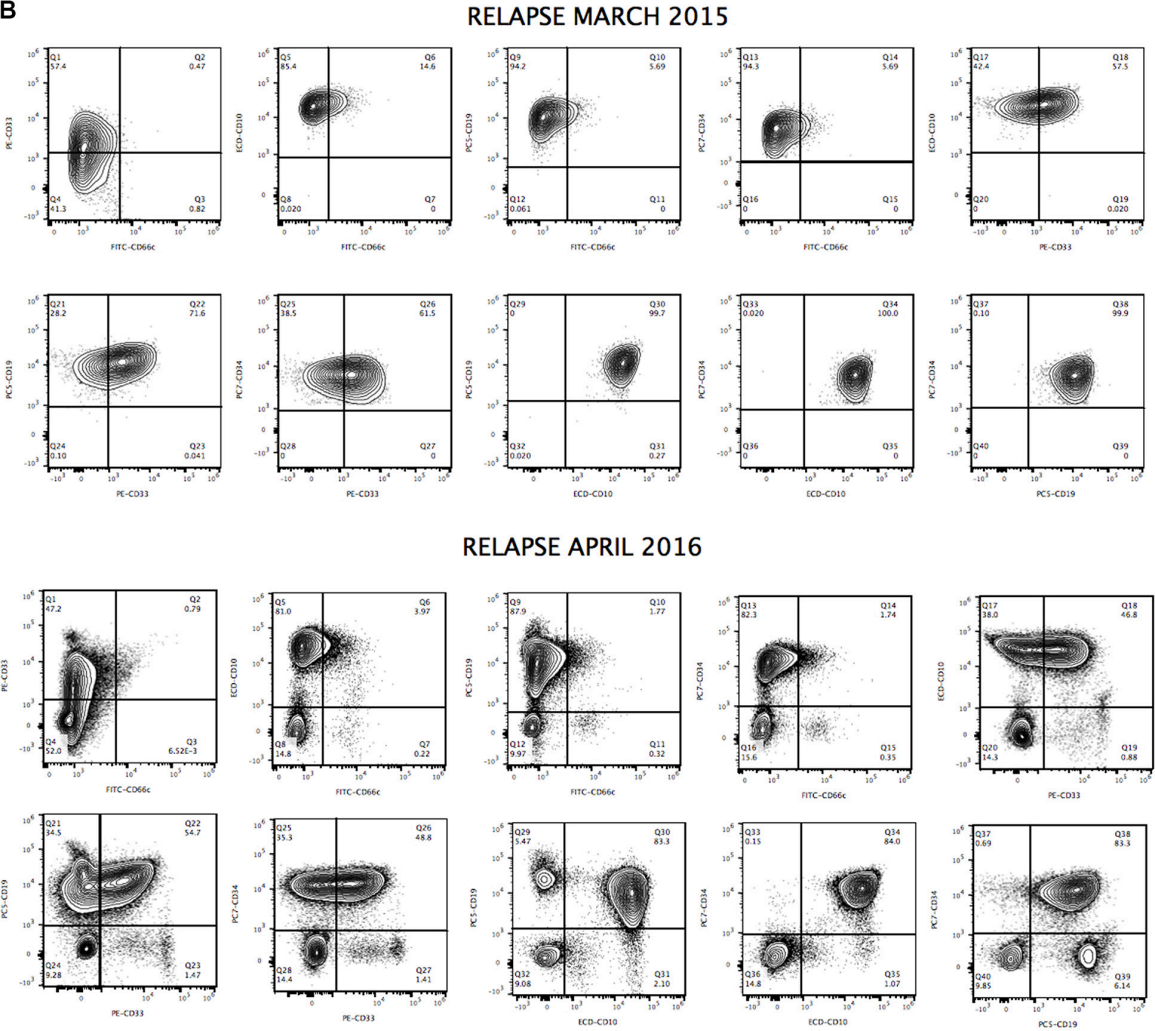
Apparently clonal populations observed at diagnosis and during relapse (Case 1) Immunophenotyping of B-ALL CD34/CD10 and CD34 CD19 cells used to identify cell populations at diagnosis and during relapse is displayed in panel (**A**), showing significant expansion of clonal CD34+ cells detected in the blood of patient 1 at diagnosis and during relapse. Characterization of apparently clonal B-cells by simultaneous combination of CD10, CD19, CD33, CD34 and CD66c at second and third relapse. Contour plots show the distribution of detected B-ALL cells, illustrating antigenic density for each marker and displayed in panel (**B**). Upper and lower panels display comparative phenotypes at second and third relapse respectively. ALP activity test allows to better discriminate subsets of apparently clonal leukemic cells based on CD10/CD19/CD33/CD34/CD66c positive expression.

The presence of apparently clonal populations does not necessarily represent clonal neoplasia. Figures 5A and 5B show significant expansion of clonal CD34+ cells detected in the blood of patient 1 at diagnosis and during relapse. However, ALP activity allows better discrimination of subsets of apparently clonal CD34+ cells, as shown in Figure 2. A phenotype of apparently clonal leukemic cells was based on CD10/CD19/CD33/CD34/CD66c positive expression. One possible explanation for the amplification of apparently monoclonal leukemic cells is clonal evolution, and preexisting resistant subclones could also explain cases arising from very rare premalignant hematopoietic cells, constituting a potential cellular reservoir that may play a key role in continuously seeding relapsed disease. As the first patient was enrolled in a clinical study of CD19-specific CAR+ T-cell immunotherapy (June 2016), the continued monitoring of CD19 is needed, as this antigen can be down-regulated or expressed at cytoplasmic levels in malignant cells to obtain a survival benefit. Moreover epitopic changes can occur, making crucial the development of highly-compact epitope-based markers combining target epitopes from different antigens [18].

**Figure 6:**
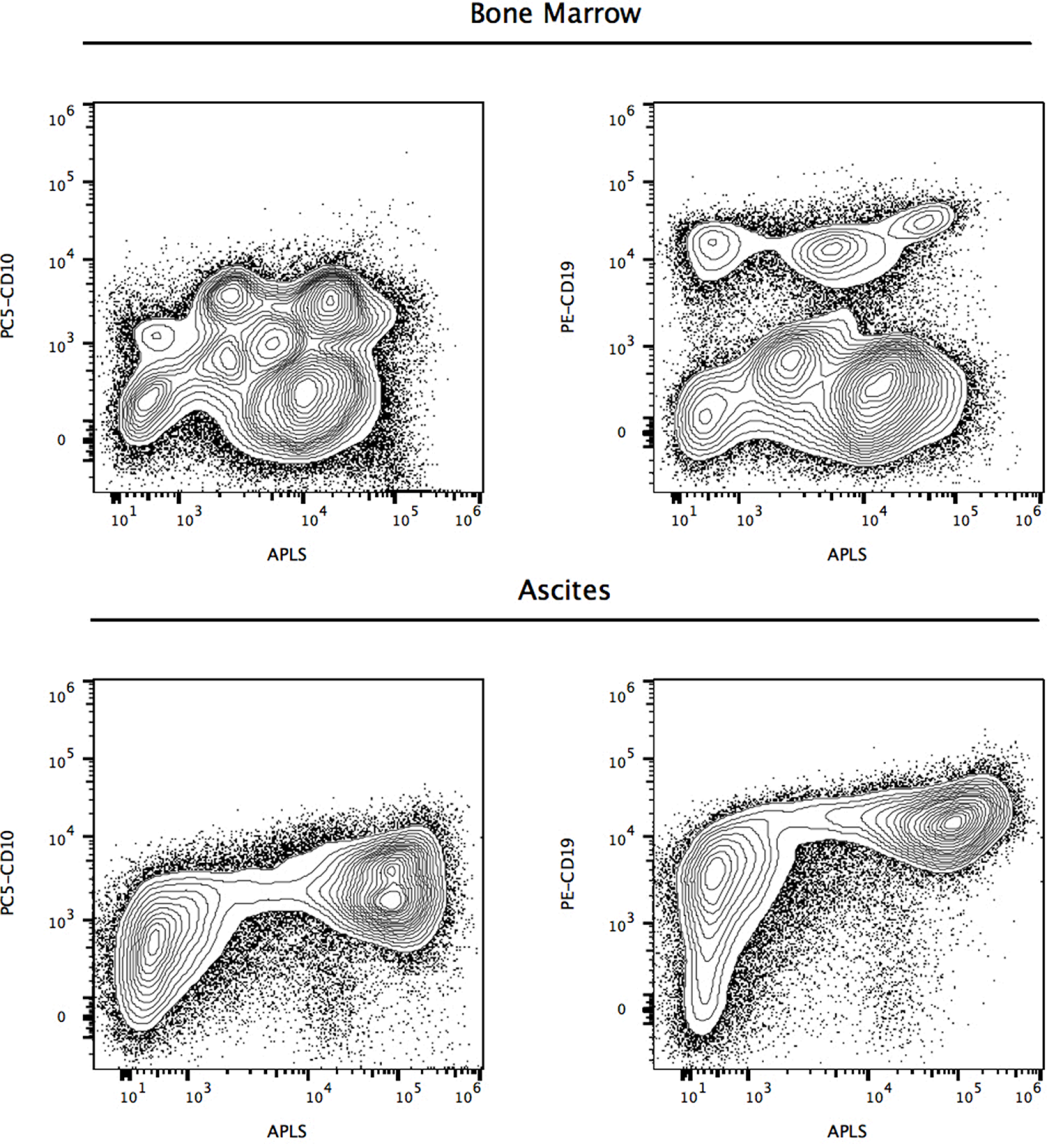
Candidate refractory CD10+/CD19+ Burkitt lymphoma cells at diagnosis expressing high levels of alkaline phosphatase activity (Case 3) Reference contour plots of two specimens obtained from the third patient, showing the performance of the alkaline phosphatase test in combination with CD10 and CD19 staining. Upper and lower panels are used to display comparison and classification of ALP+ cells, showing different subsets of CD10+ and CD19+ cells, in marrow and ascites respectively. ALP test was applied to a 12-year-old boy (Case 3) with peripheral blood immunophenotyping compatible with the diagnosis of Burkitt's lymphoma, accompanied by bone marrow aspirate showing infiltration, pleural effusion and ascites, with 15 to 87% CD10+/CD19+ cells. Cytogenetic studies showed a 46, XY karyotype, with t(8;14)(q24;q32) translocation and *C-MYC* rearrangement. Flow cytometry analyses of CD10+/CD19+/ALP+ lymphoma cells found in marrow and ascites, with increased activity of ALP in ascites lymphoma cells.

To evaluate the potential role of ALP+ cells in lymphoma, we applied our methodology to a 12-year-old boy (Case 3), with peripheral blood immunophenotyping compatible with the diagnosis of Burkitt's lymphoma, accompanied by bone marrow aspirate showing infiltration, pleural effusion and ascites, with 15 to 87% CD10+/CD19+ cells. Cytogenetic studies showed a 46, XY karyotype, with t(8;14)(q24;q32) translocation and *C-MYC* rearrangement. Flow cytometry analyses of CD10+/CD19+/ALP+ lymphoma cells found in marrow and ascites, are shown in Figure 6, evidencing an increased activity of ALP in ascites lymphoma cells.

Many experiments performed in our laboratory are functional-based assays. Upon sample reception, blood cells are immediately prepared for flow cytometry analysis in as little time as possible. Our originally developed no-lyse, no-wash protocols [19–20] allow “untouched” processing of whole blood and bone marrow by simply adding reagent, including a nucleic acid stain for identifying nucleated cells and diluting and running analysis at a higher sample rate than is practical for a conventional cytometer. Panels of immunomarkers in combination with functional analysis can provide more information than single phenotype. This combination should allow the identification and characterization of primitive progenitor cells with unprecedented levels of sensitivity for accurate and early monitoring of leukemia diagnostics and MRD. Our preliminary results suggest that CD34+/ALP^high^ cells appear to sustain leukemogenesis over time. It is unclear whether these cells can develop leukemia and we will need to learn more about some of the special features found in the biology of CD34+/ALP^high^ cells by analyzing the expression of primitive stem cell markers and their association with malignancy at a phenotypic and functional level, using a large set of leukemic malignancies and leukemic stem cells.

We also need to increase the awareness of the biological significance of increased ALP activity in leukemic refractory cells to accelerate breakthroughs in leukemia prevention, diagnosis and treatment that develop or exploit new knowledge about leukemic stem cell populations. This is a very complex and challenging task. Leukemia testing to confirm diagnosis includes complete cell counting, cytomorphology, cytogenetics and immunophenotyping studies, and molecular testing, and the number of cells available from a patient aspirate is limited. This makes the development of new assays useful for determining the basis of phenotypic and functional events associated with leukemic transformation difficult. Further experiments will be needed, such as fluorescence activated cell sorting in combination with targeted next generation sequencing studies to enlighten the role of primitive subsets of ALP^high^ cells in human leukemia and lymphoma.

## MATERIALS AND METHODS

Our no-wash no-lyse strategy uses Vybrant^®^ DyeCycle™ Violet stain (DCV), a low cytotoxicity permeable DNA-specific dye that can be used for DNA content cell cycle analysis and stem cell side population by flow cytometry. DCV can be excited with violet 405 nm laser light and can be used for simultaneous staining with alkaline phosphatase live stain (APLS). APLS can be excited at 488 nm and its emission can be collected using a standard FITC filter (for example 530/30). This protocol is ideally suited to study the numbers of ALP+ cells and it takes advantage of classic methods that are widely used in fluorescence microscopy. It avoids lysis and centrifugation steps, which can result in unwanted damage to leukocytes and CD34+ cells. Moreover, by moving PE-CD34 to the yellow laser, there is no spectral overlap between APLS and PE, eliminating the need to perform compensation and thus simplifying the experimental design and instrument set up. For instruments upgraded with a yellow laser kit, PE-CD34 is excited at 561 nm and its emission is collected in the YL1 detector (Attune NxT, Thermo Fisher Scientific, Eugene, Oregon, USA).

We previously verified the reproducibility of the new assay and data were immediately acquired after staining. Leukemic cells were incubated in the presence of APLS for 20 min at 37°C and subsequently were stained with PE-CD34 antibody for 20 min at room temperature as recommended by the manufacturer.

### Samples

Human blood samples were obtained from samples taken for clinical testing. Peripheral blood and bone marrow specimens were used, and detailed information for the analysis of leukemic cells is provided. All samples were processed immediately after collection. All procedures were performed in accordance with the internal protocols of our laboratory, which were authorized by the HUGTiP Ethical Committee, in accordance with current Spanish legislation, by the Departament de Medi Ambient i Habitatge (file #1899) of the Autonomous Government of Catalonia (Generalitat de Catalunya).

### Materials

Vybrant^®^ DyeCycle™ Violet Stain, Alkaline Phosphatase Live Stain (500×), and PE-CD34 were obtained from Thermo Fisher. Hanks′ Balanced Salt Solution (HBSS), no calcium, no magnesium, no phenol red, was obtained from PAA Laboratories, Austria.

### Flow cytometry

The Attune flow cytometer was switched on and allowed to warm up for 15 minutes before analyses were begun. Attune software was started and waste, focusing fluid, wash and shutdown bottles were checked during this time, and emptied or refilled as required. Performance validation of the Attune was then done using fluorescent beads. Anticoagulated blood or marrow containing 5 × 10^5^ cells was diluted 1/10 in HBSS, and 100 μL of diluted blood was used for incubation for 20 min at 37°C in the presence of 1 μL of APLS and 1 μL of DCV stain, protected from light in a dedicated water bath. Following incubation 5 μL of PE-CD34 was added to cells, and incubated again for 20 min at room temperature, also protected from light. Alternatively, PC5-CD10 and PE-CD19 antibodies were added for immunostaining (Case 3). Subsequently, cell suspension was diluted again with HBSS prior to sample acquisition, 500 μL final volume. All cell measurements were done using the Attune Acoustic Focusing Cytometer and the Attune NxT Cytometer.

### Instrument configuration

Side scatter (SSC) was detected using the blue laser at 488 nm and a 488/10 bandpass filter. APLS was detected with the blue laser 488 nm excitation and a 530/30 bandpass filter in the BL1 (Blue Laser) detector. For the Attune^®^ NxT upgraded with the yellow laser kit, PE was excited at 561 nm and its emission was collected using the following filter combination: 595 LP, 600 DLP, and 585/16 BP in the YL1 detector. By moving PE to the yellow laser, there is no spectral overlap between APLS and PE, eliminating the need to perform compensation and thus simplifying the experimental design and instrument set up. PC5 was detected with the blue laser 488 nm excitation and a 695/70 bandpass filter in the BL3 detector. DCV was excited at 405 nm and its emission was collected using the following filter combination: 413 LP, 495 DLP, and 440/50 BP in the VL1 detector. APLS, PE, PC5 and DCV fluorescence are displayed in logarithmic scale.

### Sample acquisition

DCV threshold levels were set empirically using a Blue-SSC vs. DCV-H dual parameter plot to eliminate from detection the large amounts of red blood cells that are found in unlysed whole blood. A proper threshold was set at 0,3 × 1000 on the Attune NxT, and this setting also excludes from acquisition non-nucleated cells and debris. This value was adjusted while acquiring data and observing the position of the DCV+ cells on the bivariate dot plots such that all the nucleated blood cells were on scale with the least amount of debris appearing in these plots. Other specimens such as marrow may appear with different scatter properties and minor variations in fluorescence intensities based on chromatin condensation. Samples were collected first using the Violet 405 nm excitation laser for V-DCV with the Blue 488 nm excitation laser for Blue-SSC detection. Blue 488 nm excitation was also used for APLS detection and the Yellow 561 nm excitation laser was used for PE detection.

## SUPPLEMENTARY MATERIALS FIGURES AND TABLES


