# Correction: Is alkaline phosphatase the smoking gun for highly refractory primitive leukemic cells?

**DOI:** 10.18632/oncotarget.26816

**Published:** 2019-03-19

**Authors:** Laura G. Rico, Jordi Juncà, Mike D. Ward, Jolene Bradford, Jordi Petriz

**Affiliations:** ^1^ Josep Carreras Leukemia Research Institute, Universitat Autònoma de Barcelona, Spain; ^2^ Thermo Fisher Scientific, Eugene, Oregon, USA

**This article has been corrected:** The correct affiliation information is given below:

^1^ Josep Carreras Leukemia Research Institute, Universitat Autònoma de Barcelona, Spain

Original article: Oncotarget. 2016; 7:72057-72066. https://doi.org/10.18632/oncotarget.12497

