# Supplementary material for: Is alkaline phosphatase the smoking gun for highly refractory primitive leukemic cells?
Source: Oncotarget. 2016 Oct 6;7(44):72057–66. doi: 10.18632/oncotarget.12497 (PMC5342144; doi:10.18632/oncotarget.12497)
Supplement: Supplementary file 1 [file oncotarget-07-72057-s001.pdf]

Is alkaline phosphatase the smoking gun for highly refractory primitive leukemic cells?

Supplementary Materials

Supplementary Information S1

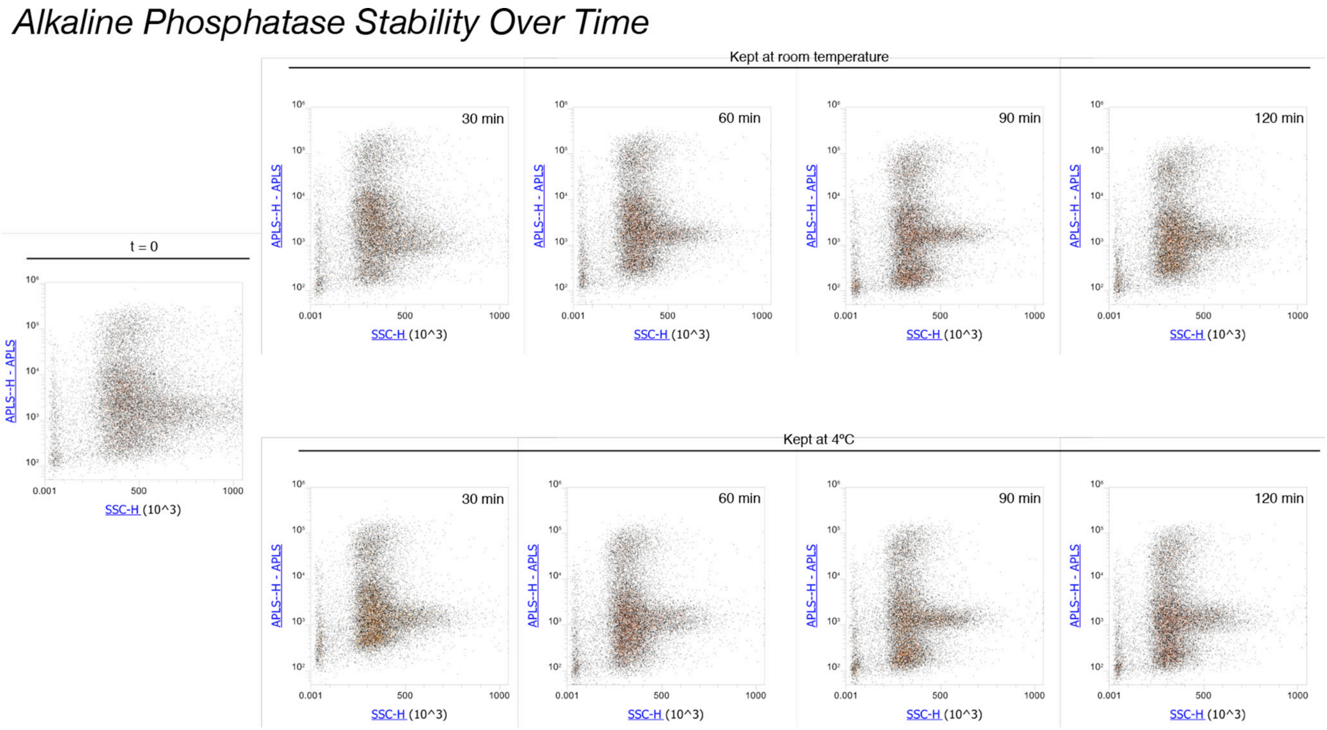

Supplementary Figure S1: Alkaline phosphatase (ALP) is a phenotypic marker of pluripotent stem cells, including undifferentiated embryonic stem cells, induced pluripotent stem cells (PSCs), and embryonic germ cells. While ALP is expressed in most cell types, its expression is highly elevated in PSCs. ALP staining has therefore been used to differentially stain PSCs to easily distinguish them from mouse embryonic fibroblasts used as feeders and parental fibroblasts commonly used in reprogramming experiments. However, current available alkaline phosphatase substrates are toxic to the cells, which prevent them from propagating once stained. The Alkaline Phosphatase Live Stain can be applied to blood and marrow specimens with minimal background in flow cytometry, providing an easy-to-use, live monitoring method to track cells in combination with immunophenotyping. Fluorescently labeled cells keep their signal up to 120 minutes even at 4ºC or atroom temperature. Our no-wash no-lyse strategy uses Vybrant® DyeCycle™ Violet stain (DCV), a low cytotoxicity permeable DNA-specific dye that can be used for DNA content cell cycle analysis and stem cell side population by flow cytometry. DCV is excited with violet 405 nm laser light and can be used for simultaneous staining with alkaline phosphatase live stain (APLS). APLS is excited at 488 nm and its emission is collected using a standard FITC filter (for example 530/30). This protocol was used to study the ALP+ fluorescence stability over time.

Supplementary Information S2

Case 1: Immunophenotyping at diagnosis (I)

Three-color analysis of MPO, CD79a, and CD3

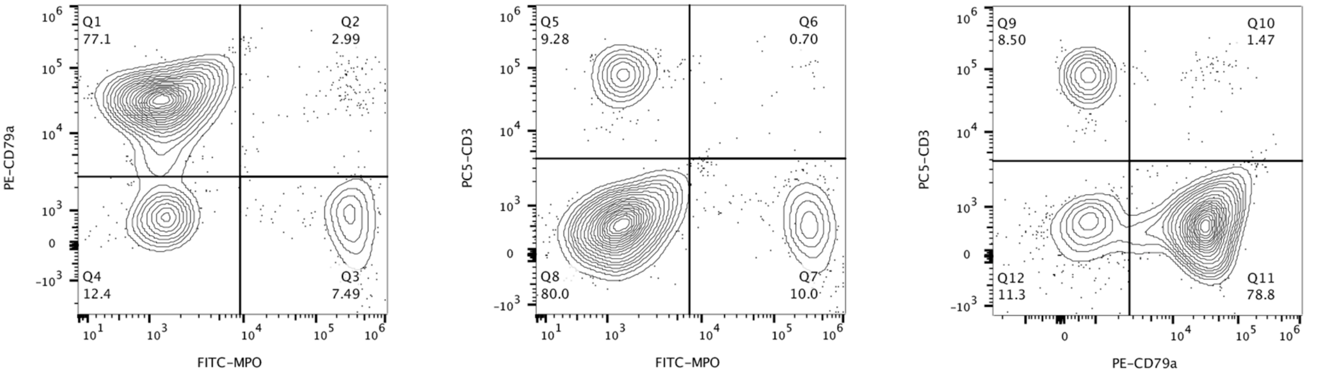

Three-color analysis of TdT, IgM, and CD3

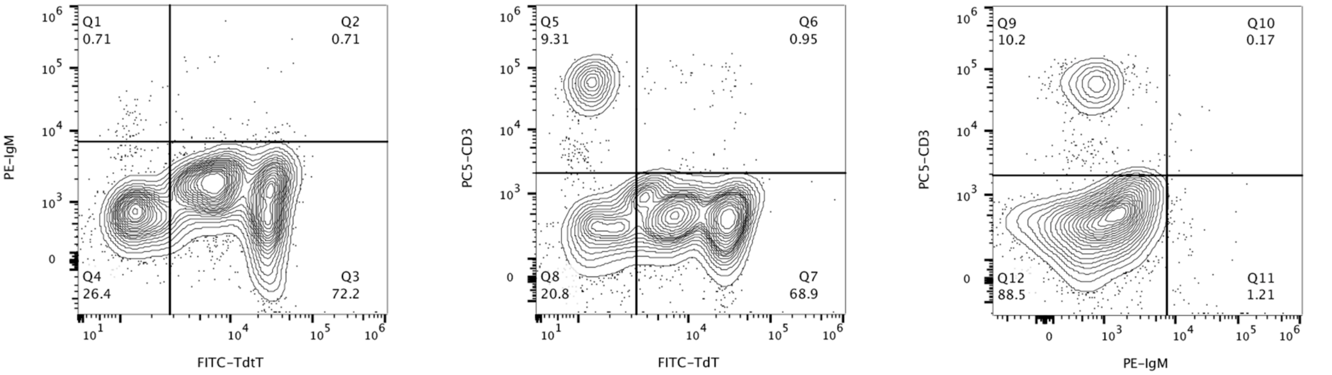

Three-color analysis of Lysozyme, CD34, and CD3

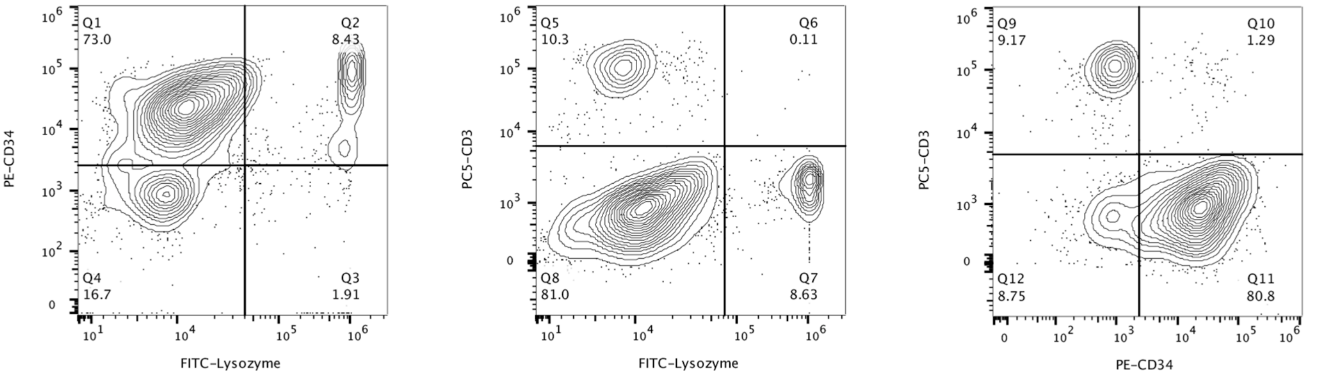

Case 1: Immunophenotyping at diagnosis (II)

Four-color analysis of CD22, CD19, CD34, and CD38

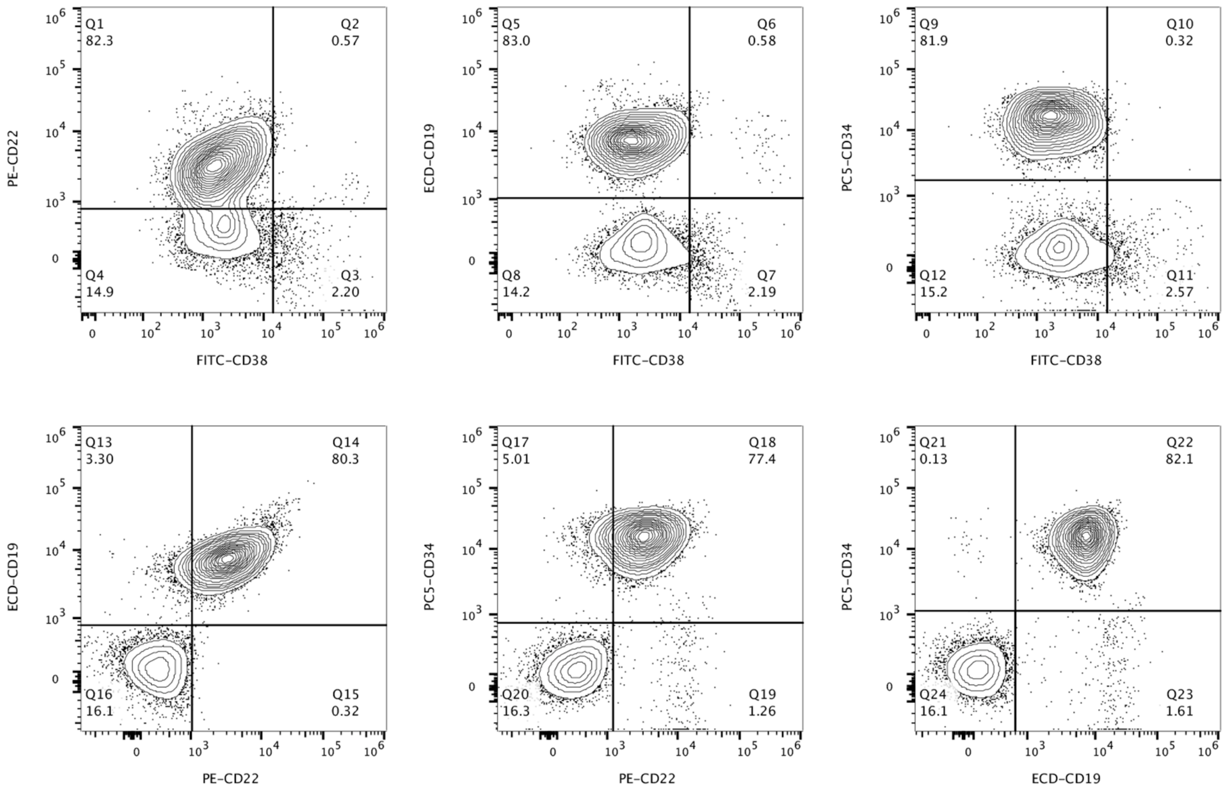

Four-color analysis of CD19, CD33, CD66c, and CD45

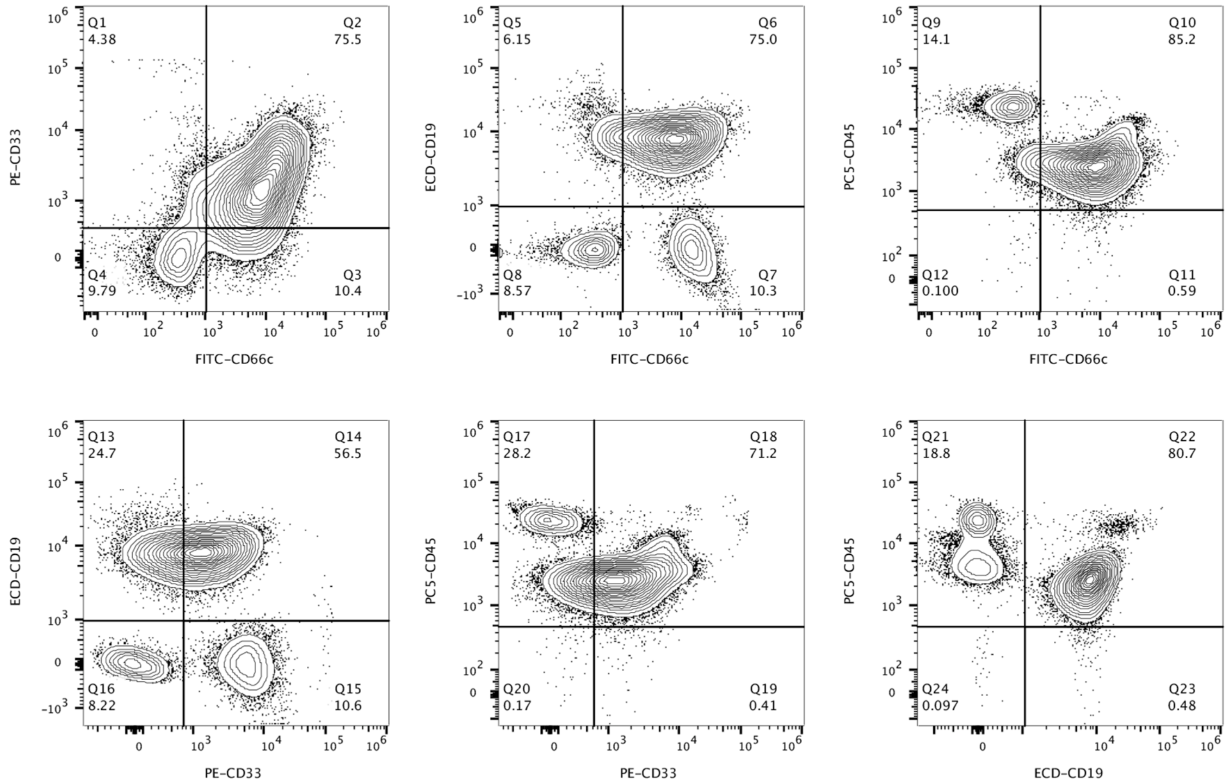

Case 1: Immunophenotyping at diagnosis (III)

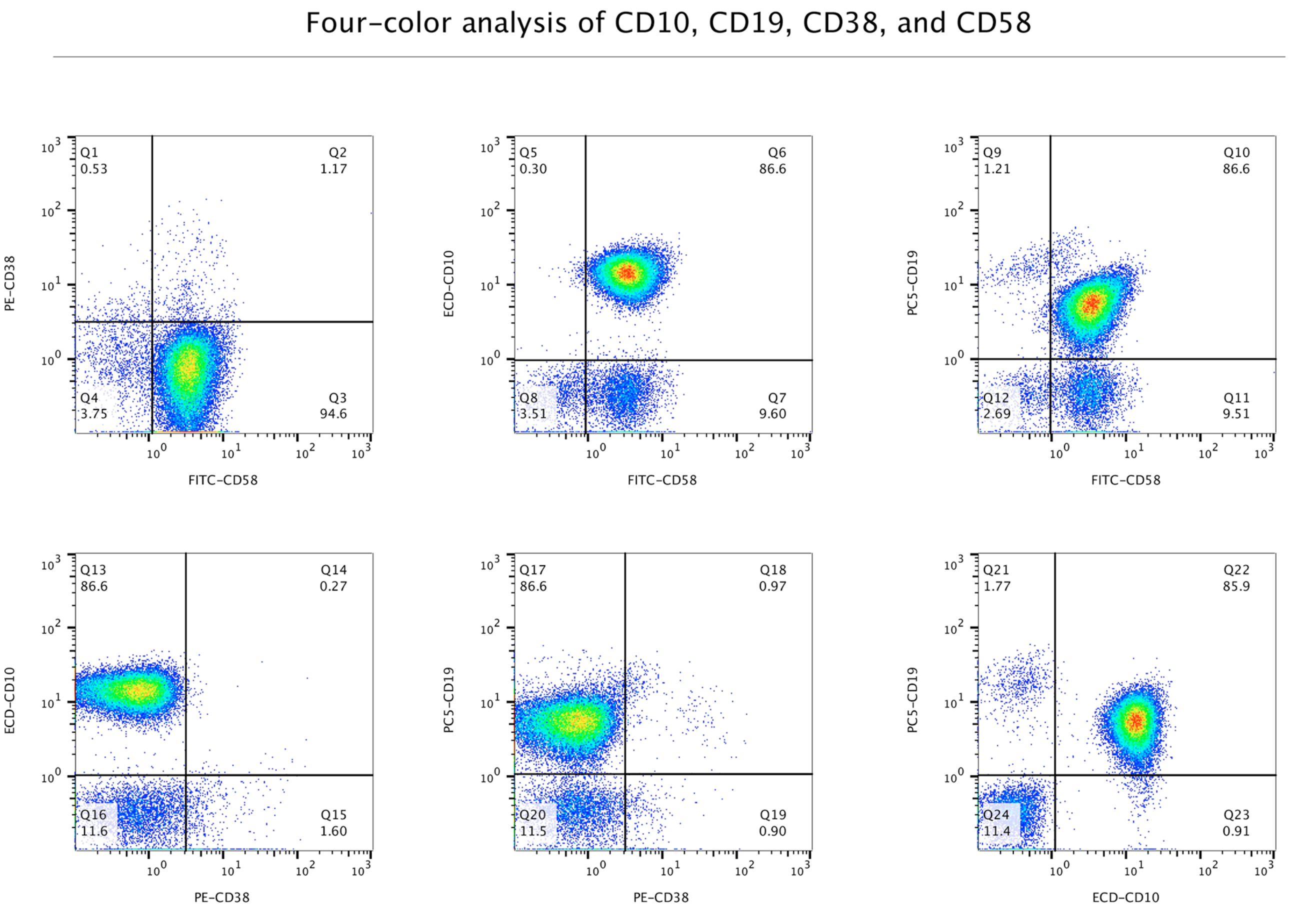

Case 1: Immunophenotyping at relapse (I)

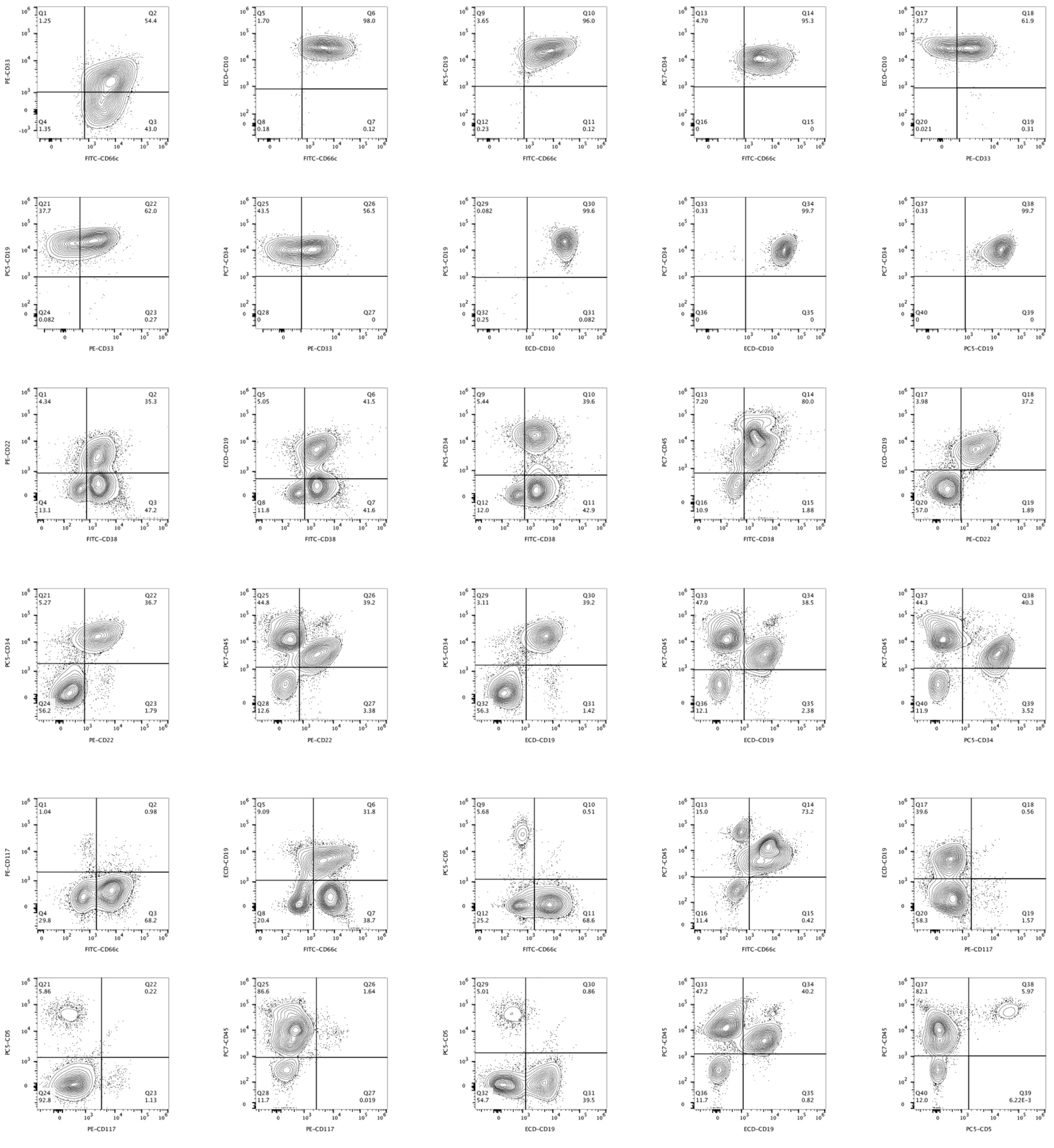

Case 1: Cell Cycle analysis at follow up after 2nd relapse, at 3rd relapse and after 3rd relapse.

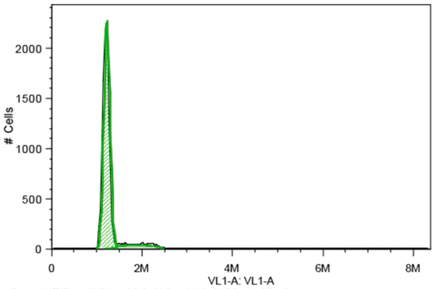

Dean/Jett/Fox: %G1 = 88.6; %S = 11.9; %G2 = 1.27e-3.
Cell Cycle
Specimen IJCBM20(2) Tween TIME.fcs
Event Count: 13712

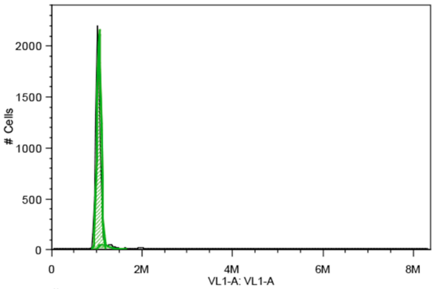

Dean/Jett/Fox: %G1 = 89.2; %S = 9.24; %G2 = 1.28.
Cell Cycle
Specimen IJCBM20(3) EtOH.fcs
Event Count: 8126

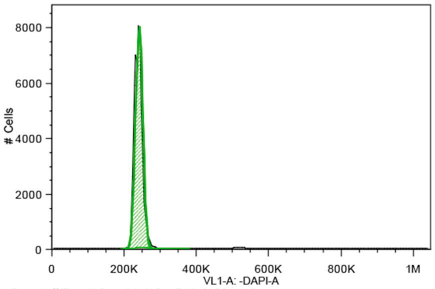

Dean/Jett/Fox: %G1 = 94; %S = 5.13; %G2 = 4.24.
Cell Cycle
BM20(4) TRITON.fcs
Event Count: 53364

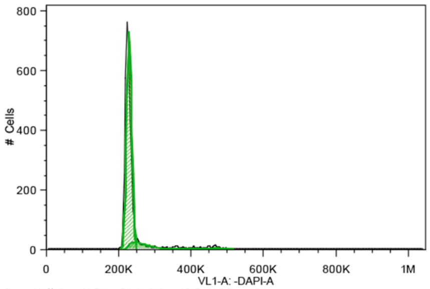

Dean/Jett/Fox: %G1 = 80.2; %S = 19.9; %G2 = 1.24.
Cell Cycle
BM20(5) TRITON.fcs
Event Count: 3726

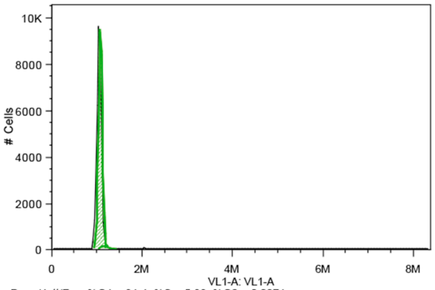

Dean/Jett/Fox: %G1 = 91.4; %S = 5.33; %G2 = 0.0271.
Cell Cycle
Specimen IJCPB35 EtOH.fcs
Event Count: 34222

Supplementary Information S3

Case 2: Immunophenotyping at diagnosis (I)

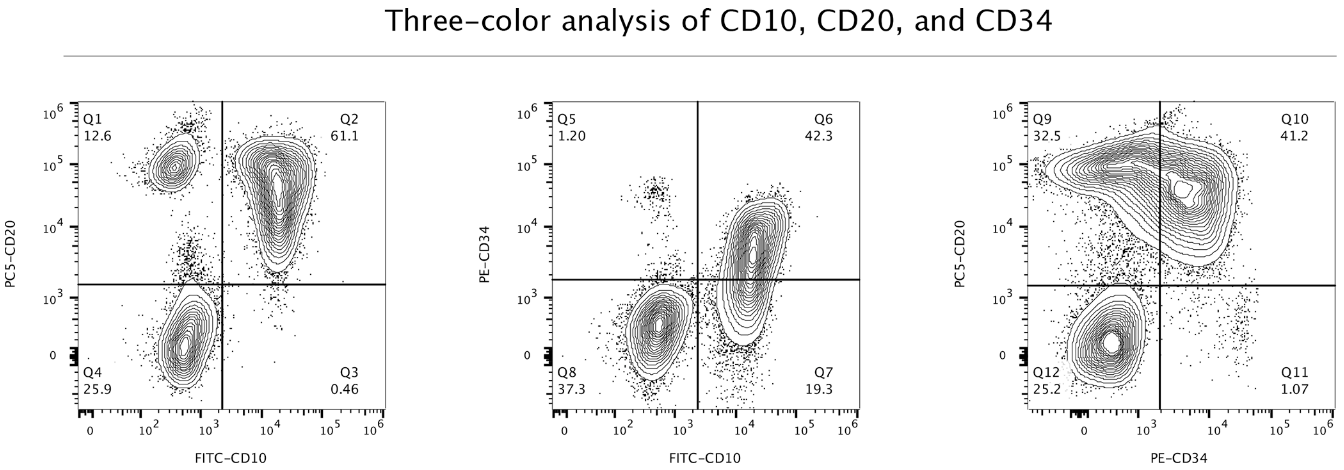

Case 2: Immunophenotyping at diagnosis (II)

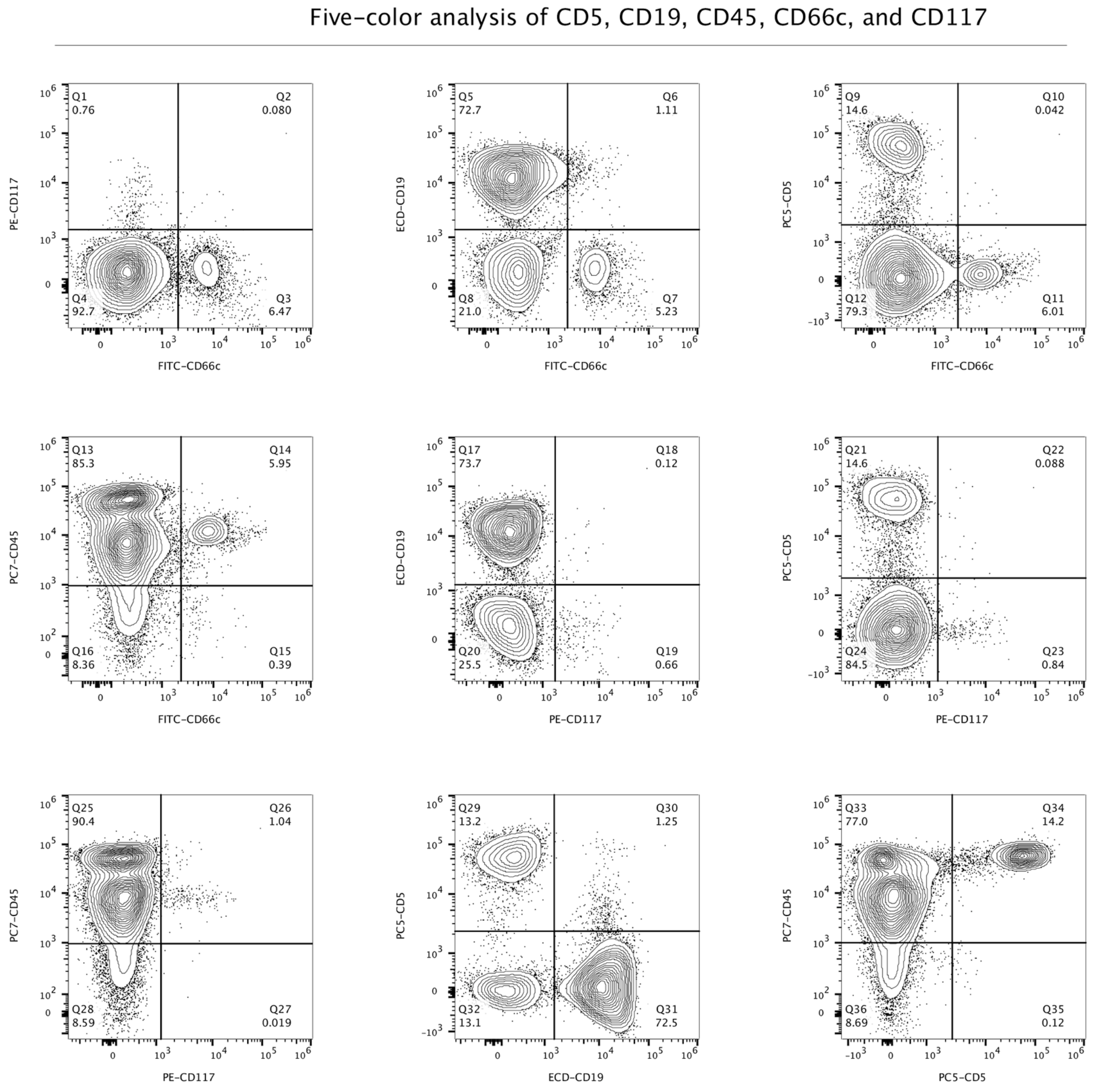

Case 2: Immunophenotyping at diagnosis (III)

Three-color analysis of MPO, CD79a, and CD3

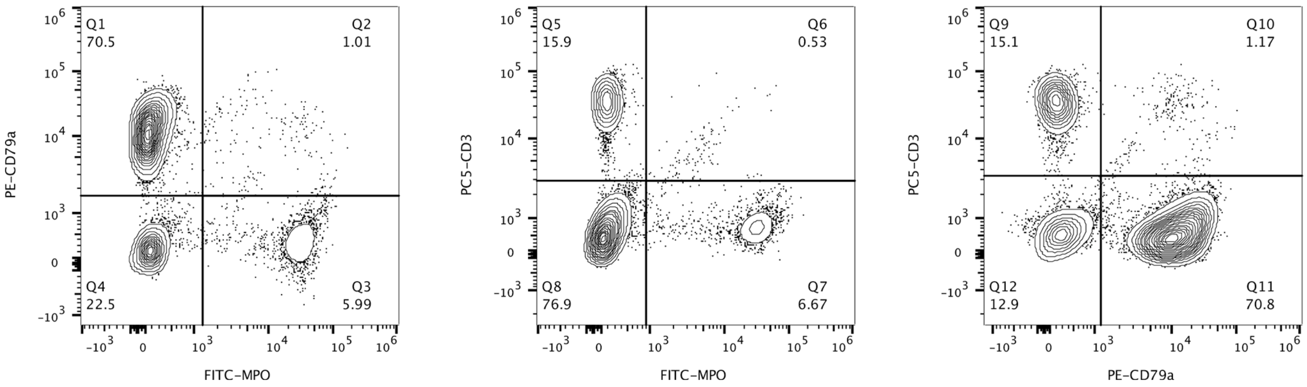

Case 2: Immunophenotyping at diagnosis (IV)

Three-color analysis of TdT, IgM, and CD3

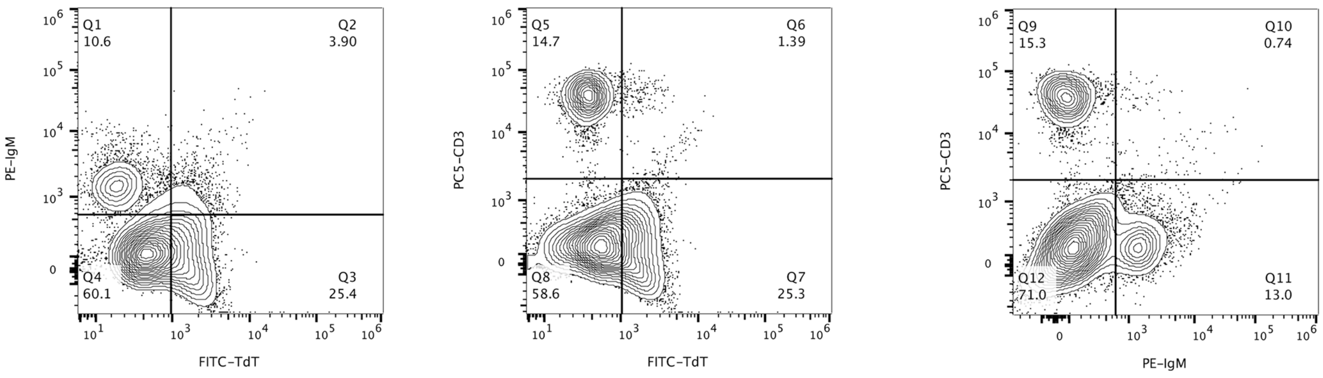

Supplementary Information S4

Alkaline phosphatase live stain for stem cell research

Background

Alkaline phosphatase (AP) is a phenotypic marker of pluripotent stem cells (PSCs), including undifferentiated embryonic stem cells (ESCs), induced pluripotent stem cells (iPSCs), and EGCs. While AP is expressed in most cell types, its expression is highly elevated in PSCs.

AP staining has therefore been used to differentially stain PSCs to easily distinguish them from mouse embryonic fibroblasts (MEF) used as feeders and parental fibroblasts commonly used in reprogramming experiments. However, current available alkaline phosphatase substrates are toxic to the cells, which prevent them from propagating once stained.

Since most of stem cells express multidrug resistance transporters, we have validated APLS as a marker for stem-like Side Population cells.

Flow Cytometry

Data were collected using the Attune Acoustic Focusing Cytometer (Thermo Fisher Scientific) equipped with two lasers operating at 405 and 488 nm.

Filter combination for APLS analysis was: 555 DLP, BP 530/30 (green). DCV signal was measured using linear scale at 50 mW. APLS was measured using logarithmic scale at 20 mW.

Acquisition was stopped when 100,000 live-gated events were collected. Propidium iodide was collected through a band pass filter of 630/30. Gating was based on forward scatter and side scatter dot plots, by encircling populations with amorphous regions and then excluding dead cells (life-gate) by propidium iodide counter-staining.

Alkaline Phosphatase Staining general procedure:

1. Remove the growth medium from the cultures to be stained with AP Live Stain.
2. Wash the culture with pre-warmed DMEM/F-12 for 2–3 minutes. Aspirate and repeat.
3. Prepare a 1X AP Live Stain by diluting the 500X stock solution in DMEM/F-12.
4. Incubate culture for 20 minutes.
5. Remove the AP Live Stain and wash twice with DMEM/F-12 for 5 minutes per wash.
6. Add fresh medium prior to the visualization of labeled cells using a standard FITC filter.

For simultaneous staining of SP cells, APLS is added for the last 20 minutes of incubation. SP cells are analyzed as described in Flow Cytometry of the Side Population, Current Protocols in Cytometry 9.23.1-9.23.20, April 2013.

Violet laser power less than 50 mW may be suboptimal for the SP studies. As a result, both blue and red linear CVs are higher with poor quality for the red emission.

When Vybrant® DyeCycle Violet stain is excited at 488 nm there is a low-level green to orange fluorescence spillover emitted by DNA-bound, and limits the use of fluorescein, PE, and PE-Cy5. APC, PE-Cy7, and APC-Cy7 in combination with red laser excitation are preferred for polychromatic measurements. However, no fluorescence spillover was observed when APLS was used in combination with DCV.

RESULTS

Alkaline phosphatase live stain is not a substrate for ABCG2

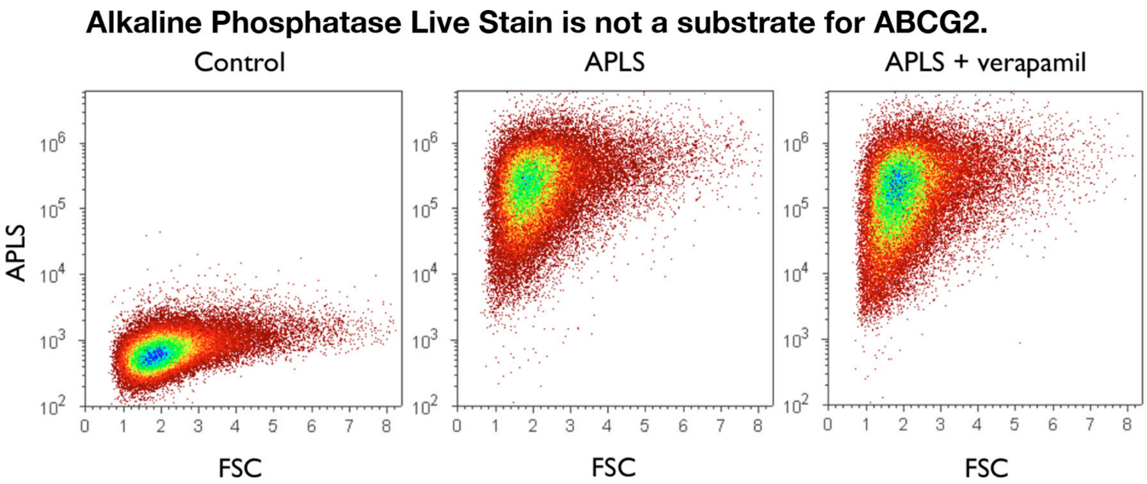

Supplementary Figure S2: KB cells transfected with the full-length ABCG2 cDNA (R482 variant; MXRA cells) were incubated with APLS in presence or in absence of the ABCG2 inhibitor verapamil for 1 hour, showing that APLS is not effluxed by multidrug resistance pumps, making feasible this stain for the identification of differentially expression within stem-like Side Population cells.

Alkaline phosphatase is expressed differentially under normoxic and hypoxic conditions

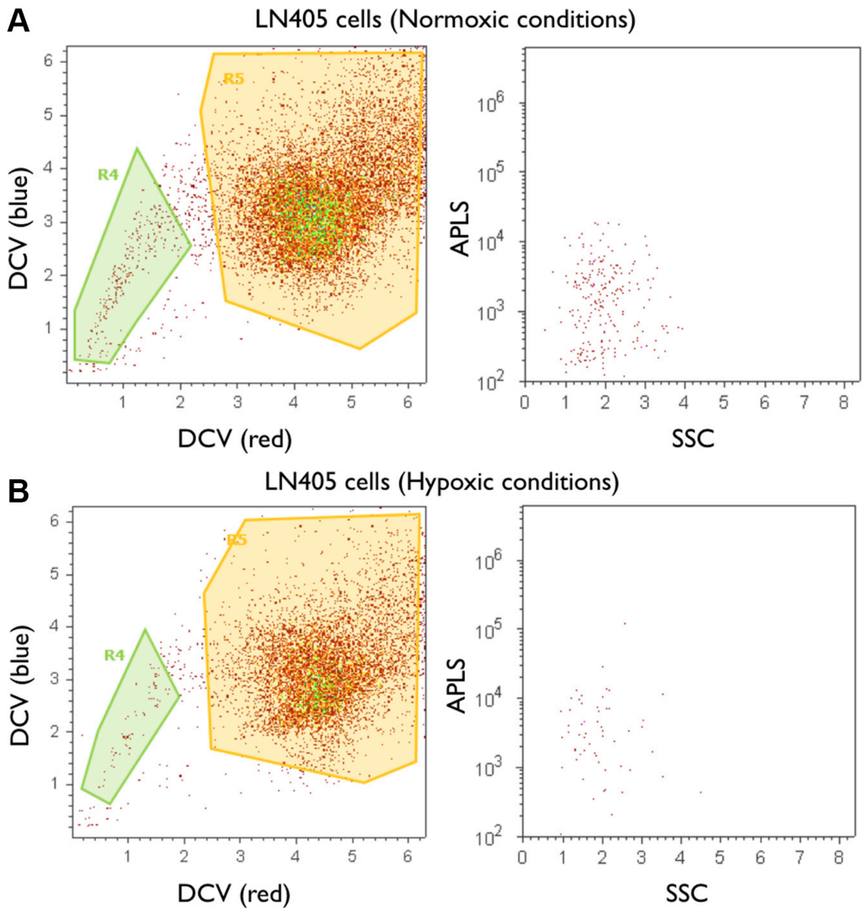

Supplementary Figure S3: LN405 cells were grown under normoxic (21% O_2) and hypoxic (5% O_2) conditions (A and B respectively) and were stained as described for SP analysis. APLS was added for the last 20 minutes of SP incubation, showing that alkaline phosphatase is overexpressed when SP cells were incubated at low O_2 concentrations.
